# A comparative, randomised MRI study of the physiological and appetitive responses to gelling (alginate) and non-gelling nasogastric tube feeds in healthy men

**DOI:** 10.1017/S0007114523000302

**Published:** 2023-10-28

**Authors:** Abdulsalam I. Aliyu, Aline Nixon, Caroline L. Hoad, Luca Marciani, Maura Corsetti, Guruprasad P. Aithal, Sally M. Cordon, Ian A. Macdonald, Maha H. Alhussain, Hiroaki Inoue, Masahiko Yamada, Moira A. Taylor

**Affiliations:** 1 Department of Human Physiology, College of Medical Sciences, Gombe State University, Gombe, Nigeria; 2 The David Greenfield Human Physiology Unit, Division of Physiology, Pharmacology and Neuroscience, School of Life Sciences, Faculty of Medicine and Health Sciences, University of Nottingham, Nottingham, UK; 3 Sir Peter Mansfield Imaging Centre, School of Physics and Astronomy, University of Nottingham, Nottingham, UK; 4 NIHR Nottingham Biomedical Research Centre at Nottingham University Hospitals NHS Trust and University of Nottingham, Nottingham, UK; 5 Nottingham Digestive Diseases Centre, Translational Medical Sciences, School of Medicine, University of Nottingham, Nottingham, UK; 6 Nestlé Institute of Health Sciences, Nestlé Research, Société des Produits Nestlé S.A, Lausanne, Switzerland; 7 Department of Food Science and Nutrition, King Saud University, Riyadh, Saudi Arabia; 8 Global Planning Group, Medical Solutions Vehicle, KANEKA CORPORATION, Osaka, Japan; 9 Regenerative Medicine and Cell Therapy Laboratories, KANEKA CORPORATION, Kobe, Japan

**Keywords:** Alginate, Gelling Nasogastric feed, Diarrhoea, MRI

## Abstract

Inclusion in nasogastric tube feeds (NGTF) of acid-sensitive, seaweed-derived alginate, expected to form a reversible gel in the stomach, may create a more normal intragastric state and modified gastrointestinal responses. This may ameliorate NGTF-associated risk of diarrhoea, upper gastrointestinal symptoms and appetite suppression. In a randomised, crossover, comparison study, undertaken in twelve healthy males, an alginate-containing feed (F + ALG) or one that was alginate-free (F-ALG) (300 ml) was given over 1 h with a 7–14-d washout period between treatments. Baseline and for 4-h post-feed initiation, MRI measurements were made to establish small bowel water content (SBWC), gastric contents volume (GCV) and appearance, and superior mesenteric artery blood flux. Blood glucose and gut peptides were measured. Subjective appetite and upper gastrointestinal symptoms scores were obtained. *Ad libitum* pasta consumption 3-h post-feeding was measured. F + ALG exhibited a gastric appearance consistent with gelling surrounded by a freely mobile water halo. Significant main effects of feed were seen for SBWC (*P* = 0·03) and peptide YY (PYY) (*P* = 0·004) which were attributed to generally higher values for SBWC with F + ALG (max difference between adjusted means 72 ml at 210 min) and generally lower values for PYY with F + ALG. GCV showed a faster reduction with F + ALG, less between-participant variation and a feed-by-time interaction (*P* = 0·04). Feed-by-time interactions were also seen with glucagon-like-peptide 1 (GLP-1) (*P* = 0·02) and glucose-dependent insulinotropic polypeptide (GIP) (*P* = 0·002), both showing a blunted response with F + ALG. Apparent intragastric gelling with F + ALG and subsequent differences in gastrointestinal and endocrine responses have been demonstrated between an alginate-containing and alginate-free feed.

An estimated 25 % of adults admitted to hospital in the UK are undernourished^(1)^ and are commonly treated by nasogastric tube feeding (NGTF)^(1)^. NGTF-associated diarrhoea, however, occurs in 2 % to 63 % of cases; the range being attributed to such factors as method of measurement and definition of diarrhoea^(2–7)^. Negative clinical consequences included compromised nutrient, electrolyte and fluid absorption, and increased fecal incontinence predisposing to loss of skin integrity and pressure sore infection^(8–10)^. Nausea, vomiting and upper gastrointestinal symptoms are also increased during NGTF, increasing the risk of aspiration pneumonia and death^(6,7,11)^. NGTF have also been implicated in suppressing appetite. These complications may lead to suboptimal feeding rates or withdrawal of the feed^(8)^.

Disruption to the normal gastrointestinal response to feeding may occur when feeding a liquid, via a tube, directly into the stomach. The oral and oesophageal response are bypassed, and gastric emptying rates of liquids, meals of mixed consistency, and solids into the small intestine differ^(12–14)^. This may contribute to NGTF complications with disruption of the complex central and peripheral neural, endocrine and microbial regulatory systems controlling not only gastric emptying but also freely mobile small bowel water content (SBWC)^(4–6,12–16)^. Whilst products of digestion and up to 90 % of the ingested and secreted water are usually absorbed within the small intestine, abnormal levels of small bowel water have been noted in diseases where diarrhoea is present^(17–19)^, and the rate of water delivery to the colon has been associated with fecal consistency by some^(20)^, but not others^(21)^. Furthermore, altered colonic water absorption has been identified with NGTF^(5)^. A liquid diet may increase the risk of upper gastrointestinal symptoms^(22–28)^.

Alginates, polysaccharides derived from the cell walls of brown seaweed^(29–31)^, are liquid in neutral and alkali conditions and, hence, can be intragastrically fed via a fine bore tube but would be expected to form a reversible gel in the acid environment of the stomach^(32–34)^. Formation of a gel may induce an intragastric state which is comparable to that following consumption of a semi-solid or solid meal and may reduce NGTF complications such as reflux and diarrhoea and thus is used in Japan^(32–34)^. Prior to studying the use of gelling NGTF in patient groups, it is important first to demonstrate in healthy participants, a difference in gastrointestinal response, when compared with a non-gelling NGTF.

The purpose of this study was thus to test the hypothesis, in healthy participants, that acute exposure to an NGTF that forms a gel intragastrically results in different gastrointestinal responses compared with a non-gelling feed. Using MRI, the gastric appearance, the liquid and semi-liquid gastric contents volume (GCV) and SBWC (primary outcome) were measured when feeding a gelling and non-gelling feed. SBWC was defined as freely mobile water with a bright-white appearance with MRI. Post-feeding blood glucose and serum insulin profiles, plasma gut hormone profiles, superior mesenteric artery (SMA) blood flux, and measures of upper gastrointestinal symptoms and appetitive response were obtained to identify potential mechanisms and clinical consequences. The feeds that were compared were a sodium alginate-containing feed that was expected to gel in the stomach and a non-gelling feed of similar energy and macronutrient composition.

## Materials and methods

### Participants and ethics

This study was conducted according to the guidelines laid down in the Declaration of Helsinki, and all procedures involving human subjects/patients were approved by the Faculty of Medicine and Health Sciences Research Ethics Committee of the University of Nottingham: ref: 97–1809. The study was registered with clinicaltrials.gov (ref: NCT04113200) (URL- www.clinicaltrials.gov). Written informed consent was obtained from all participants both before screening and prior to the main study. Recruitment and the main study intervention were conducted in the David Greenfield Human Physiology Laboratory and the Sir Peter Mansfield Imaging Centre of the University of Nottingham, UK. Recruitment was via posters displayed at the University of Nottingham between December 2018 and March 2020, and participants were enrolled by the lead researcher (AIA). Inclusion criteria were healthy males aged 18–45 years, with a BMI of 18·5–24·5 kg/m^2^, or a BMI of 24·5–26 kg/m^2^ and a waist circumference of less than 94 cm, and the ability to provide informed consent. Exclusion criteria were as follows: a history of acute illness lasting more than 1 week in the 6 weeks prior to starting the trial; a history of gastrointestinal disorders, including gastro-oesophageal reflux disease, irritable bowel syndrome or active peptic ulcer disease; taking medication for gastrointestinal disorders (including acid suppressants, antispasmodics and antidepressants) or medication for diabetes; a history of substance abuse in the last 6 months; previous surgery to the gastrointestinal or biliary system; symptoms of clinical depression defined by a score of > 10 on the Beck Depression Inventory^(35)^ and those with characteristics of eating disorder defined by a score of > 20 on the Eating Attitude Test – EAT-26^(36)^; and any factors that precluded safe MRI use. Volunteers were also excluded if they had allergies or an intolerance to any of the ingredients contained in the two NGTF or the cheese and tomato pasta meal, or if they had been involved in another trial less than 3 months prior to this study.

### Study design

A comparative, randomised study was undertaken with a crossover design. Each participant attended a screening visit (1 h), and then for each of the two arms of the study, a pre-study visit (30 min), an approximately 6-h study day visit (when 300 ml of nasogastric feed was administered over 1 h, with follow-up measurements over the subsequent 3 h), and a post-study day visit (30 min), 3 d later. The intervention feed, Mermed One, TERUMO CORPORATION, Japan (invented by KANEKA CORPORATION) contained sodium alginate as the main source of gelling fibre with polydextrose (a slowly fermented, non-gelling, synthetic glucose polymer)^(37)^ and cellulose (a glucose polymer with a high degree of polymerisation) (F + ALG). The comparator feed (without alginate) was Nutricomp Soy Fibre, B. Braun, Melsungen, Germany, and contained inulin (a fermentable fructan) and wheat dextrin (a fermentable, resistant starch) as sources of fibre (F-ALG). Table 1 provides a summary of the composition of the feeds, and more detail is provided in Supplementary Item 1.

**Table 1.** Composition of the two feeds per 300 ml

Per 300 ml of feed	F + ALG	F-ALG
Energy (kJ)	1255	1255
Energy (kcal)	300	300
Protein (g)	12·0	11·7
Fat (g)	11·4	10·5
Carbohydrate (g)	40·8	39·9
Fibre (g)	3·9 (alginate, polydextrose, cellulose)	4·5 (inulin, wheat dextrin)
Osmolarity (mOsm/l)	353	230

F + ALG, alginate-containing feed; F–ALG, alginate-free feed

### Randomisation and blinding

Participants were randomly assigned with equal probability to receive either feed on the first study day visit, based on a computer-generated random code (randomisation.com) by the research nurse. The alternate feed was then provided on the second visit. The lead researcher, MRI operator, participant and laboratory technician undertaking the blood analysis were blind to the feed given. The research nurse who set up the NGTF and collected the blood samples was aware of treatment sequence, for safety reasons, but had no subsequent access to data. Participants were blind to the feed provided.

### Pre-study

For 4 d prior to the study day visit, participants were instructed to: follow two repetitions of a standardised 2-d menu based on a self-completed 3-d food record that had been adjusted to provide a comparable percentage energy from macronutrients and relative fibre content to the NGTF (based on the mean content of the two feeds); refrain from strenuous physical activity; and not consume alcohol and caffeine. For the 3 d prior to the study visit, participants were instructed to collect the first stool of the day (using the kit provided) and for every stool to complete the King’s Stool Chart^(38)^ and photograph each stool using the disposable camera provided (results not reported in this paper).

### Study day visit protocol

Participants attended the MRI centre, at the Sir Peter Mansfield Imaging Centre, at 8 am having fasted overnight. Following weighing, an intravenous cannula was inserted in a retrograde direction into a dorsal foot vein for the collection of blood samples. A small intradermal injection of 1 % lidocaine was administered at the site of insertion. A 3-way tap and primary giving set were attached to the cannula and an intravenous infusion of 0·9 % saline delivered slowly to keep the vein open throughout the study visit. Participants were then intubated with a 120-cm-length 8-French gauge (FG) fine bore nasogastric tube (NGP18/120L, HMC PREMEDICAL S.p.A. Italy), inserted through an anesthetised nostril as described previously^(39)^. Fifty millilitres of water were consumed during placement which was confirmed as correct by aspiration of the stomach contents and ensuring a pH of ≤ 5·5 on CE-marked pH indicator strip (GBUK Group LtdUK). The length marking on the nasogastric tube was replicated on the second study visit. Having removed the guide wire participants entered the 3T Achieva MRI scanner (Philips Medical Systems, Best) and lay flat on the bed where they remained for the subsequent 4 h. The cannulated foot was placed in a hot air box (50–55°C) to provide arterialised venous blood^(40,41)^. The scanning bed was removed from the scanner between scans.

The first MRI scan (20 min including set-up) was undertaken to determine baseline values for SBWC, SMA blood flux^(42)^ and GCV. Two baseline blood samples were obtained with a 5-min interval between them. All blood samples over the study were drawn using the three-way tap, and the first 2 ml was discarded. Prior to each blood sample, participants completed paper-based 100-mm visual analogue scale (VAS) relating to subjective appetite sensations and upper gastrointestinal symptoms.

The feed was then delivered at room temperature, via the tube, using a bespoke, MRI compatible, pressure bag system allowing constant adjustment of the drip rate so that a total of 300 ml of feed was delivered over 1 h (+ or – 6 min).

During the 1 h of feeding and subsequent 3 h, MRI scans were undertaken approximately every half-hour; blood sampling continued at 10 min (glucose) and 20 min (insulin and gut hormones) intervals and VAS (upper gastrointestinal symptoms and appetite scores) were completed at 30-min intervals, prior to blood sampling.

Four hours from the start of feeding, participants left the scanner, the intravenous cannula and nasogastric tube were removed, and they were offered a cheese, tomato and pasta meal (served at 82°C) which they were instructed to eat, with no time restriction, until they felt comfortably full as described previously^(43)^. When approximately one-quarter of the bowl of food was left, it was replaced with a full bowl to avoid prompting meal termination (‘*Bottomless bowl technique’*)^(44)^. Participants drank 200 ml of water whilst consuming the pasta meal. The full amount prepared contained about 11 000 kJ (2630 kcal) of energy, 300 g of carbohydrate (45·6 %), 82 g of protein (12·5 %) and 122 g of fat (41·8 %) and was divided into three portions. Appetite and upper gastrointestinal symptom scores using VAS were completed immediately before the meal and immediately afterwards, and the duration of eating was recorded.

Participants left the unit having been instructed to follow their standardised food menu for the remainder of that day and the subsequent 2 d and to record compliance on the form provided. A fecal collection kit was provided for sampling of the first available fecal sample on the day of the study, and on each of the next 2 d. The participants were required to record all other stools using the adapted King’s Stool Chart^(38)^ and a reusable camera (results not reported in this paper). They were instructed to complete the upper gastrointestinal symptom scores at the end of the day, just before going to bed.

Following a 7–14-d washout period consuming their habitual diet, participants repeated the previously stated protocol with the opposite feed delivered during the study visit.

A repeat trial was conducted in a participant whose blood samples could not be taken during the MRI scans due to the cannula failing, having moved from the vein into adjacent tissue. The same protocol was repeated, but without scans, in the Sir David Greenfield Human Physiology Unit, University of Nottingham, having obtained further consent and undertaken a repeat screening.

### Procedures

#### MRI measurements

The GCV measurements were acquired using two single-shot turbo spin echo (TSE) sequences with two different TE (echo time), one with 60 min and the other 300 min, each of which lasted 22 and 36 s, respectively. Each had twenty contiguous slices, which were 10 mm thick and were acquired with in-plane resolution of 1·5 mm x 1·88 mm with no gap between slices but were reconstructed to 1 × 1 pixel size. The scans were acquired in expiration breath hold. The 36-s breath hold was split into two for convenience of the participant. This imaging sequence produced a good contrast between the stomach contents and other abdominal organs.

SBWC was acquired using a previously described and validated technique applying a fat-saturated coronal single-shot turbo spin echo sequence acquiring twenty contiguous coronal slices with reconstructed in-plane resolution of 0·78 mm × 0·78 mm and slice thickness of 7 mm with no gaps between slices, TE = 400 min and TR 1159 min. Data were collected during an expiration breath hold of 23 s.

Axial, coronal and sagittal balanced turbo gradient echo bright blood localiser scans were used to position the flow measurement plane perpendicular to the SMA. The SMA blood flux was measured using a 2D gradient echo cine phase contrast MRI scan with TE = 3·3 ms, TR = 7·5 ms and flip angle 25°. Reconstructed in-plane resolution was 1·17 mm × 1·17 mm and slice thickness 6 mm, velocity encoding 140 cm/s with flow encoding through plane. This was acquired in a 14-s breath hold.

Gastric image analysis was performed, blind to the feed, using MIPAV (Medical Imaging Processing, Analysis and Visualization) software v9.0.0 ^(45)^ which is freely available. GCV was measured by manually tracing the region of interest around the liquid and solid contents within the stomach on each slice. The GCV at each time point was calculated by multiplying the surface area of the gastric content measured per slice with the thickness of the slice and then summed up to produce the total GCV in ml, and intragastric air was excluded from the region of interest.

The SBWC images were analysed using validated in-house custom written software (IDL 6·4, Research Systems Inc.)^(17,46)^. Briefly, regions of interest were drawn around the small bowel, excluding the gall bladder, bladder, colon and stomach as well as blood vessels. Voxels with an intensity above a threshold, which was determined from that person’s cerebrospinal fluid intensity, were identified as free small bowel water. The sum of these voxel volumes within the small bowel regions of interest provided an estimate of total SBWC.

The SMA blood flux data were analysed using validated manufacturer’s software (Philips QFlow software) developed to assess the blood flow in different areas of human body^(42,46–48)^. A line was drawn approximately around the SMA vessel on one image and the software, then automatically reduced the region of interest to the size and shape of the vessel and tracked it over the cardiac cycle. The software then automatically calculated the SMA blood flux (ml/s).

#### Characterisation of intragastric gel content

**Fig. 1.**
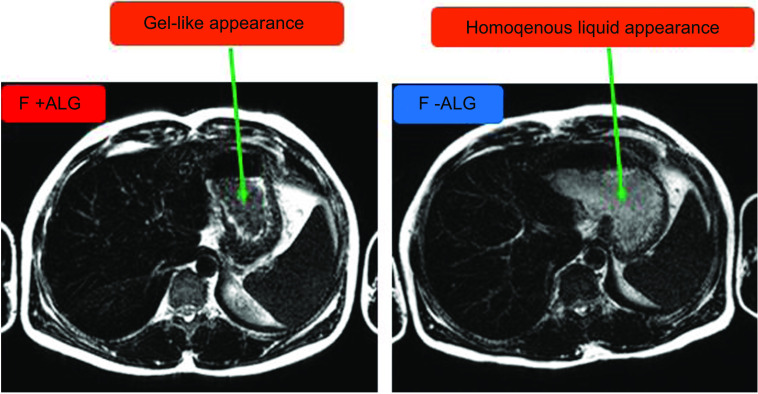
The flow of the participants from screening to final data analysis. All blood samples analysed were for seven participants except for GLP-1 (*n* 6) where one participant was excluded from analysis due to abnormally low levels which were undetectable. GLP-1, glucagon-like-peptide 1.

T2-weighted gastric images with the longer echo time (TE 300) were used to visualise and characterise the gastric contents. Images acquired were inspected for their appearance by two independent researchers with extensive experience of analysing gastric images. The images were classified into ‘consistent with gel’ and consistent with ‘non-gel’. Images were identified as having a gel-like appearance when they displayed a heterogenous intragastric dark material with a white ring around it consistent with gel formation on T2-weighted MRI imaging or non-gel with a homogenous intragastric bright material (Fig. 1). These were distinguished from air which appears uniformly darker than any aspect of the image. All slices across the time points were examined and reported. The randomisation code was then broken.

#### Blood sample collection technique, processing and analysis

For blood glucose, 0·5 ml of whole blood (WB) was collected into cold fluoride/citrate tubes, mixed gently, kept on ice and analysed within 2 h using YSI 2300 STAT PLUS (YSI Inc. Yellow Springs).

The remaining blood was gently mixed in tubes containing the appropriate preservative, centrifuged (3000 g 4°C for 10 min) and plasma/serum aspirated, aliquoted and frozen at minus 80°C as appropriate for the intended analyses. All samples were kept on ice throughout processing, except for serum samples which were kept at room temperature for the first 15 min to enable the blood to clot. Preservatives used were as follows: active glucagon-like-peptide 1 (GLP-1) required EDTA tubes with added DPP IV inhibitor (10 µl/ml WB), and total peptide YY (PYY) and total ghrelin both required EDTA tubes with added aprotinin (500 Kallikrein Inactivator units/ml WB). Insulin and total glucose-dependent insulinotropic polypeptide (GIP) required serum, and plain serum-separator tubes were used.

Analytical techniques were either RIA (PYY, ghrelin, and insulin) or ELISA (active GLP-1 and GIP) as used previously^(43,49–51)^. All kits were Merck-Millipore (EMD Millipore). For these study samples, the mean intra-assay and inter-assay CV for total ghrelin was 4·9 % and 7·7 %; PYY was 4·2 % and 9·7 %; insulin was 3·6 % and 3·8 %; active GLP-1 was 4·6 % and 9·0 % and total GIP was 2·4 % and 4·4 %, respectively.

#### VAS scores for appetite and upper gastrointestinal symptom scores

The paper-based VAS was made up of a 100 mm line anchored at each end with positive and negative extremes for each of the appetite variables (hunger, fullness, satisfaction, desire to eat and prospective food consumption). Without reference to previous ratings, participants instructed the research nurse to mark a vertical line across the horizontal 100 mm line, and the metric value (mm) was measured from the 0 mm (on the left) to the vertical mark.

The characterisation of the upper gastrointestinal symptoms scores was reported on a VAS of 0–100 mm, with absence of symptoms = 0 and very severe symptoms = 100 at each end, in conjunction with a pictorial representation of symptoms and verbal description^(52)^. The symptoms assessed were as follows: (i) how hard it was to finish a normal meal; (ii) feeling of food lying heavily in the stomach; (iii) feeling of bloating in the stomach; (iv) pain suffered in the stomach area; (v) burning feeling in the stomach area; (vi) feeling of nausea; (vii) feeling of troublesome burping; (viii) burning feeling behind breastbone; (ix) feeling of acid taste in mouth; and (x) unpleasant movement of material upwards from stomach area.

#### Sample size, data analysis and statistical analysis

The power calculation was based on the work of Marciani et al. in 2010^(17)^ who demonstrated that in eleven healthy volunteers given glucose, the mean postprandial SBWC at 40 min was 47 ml with a sd of 15 ml. We estimated that with statistical power at the level of 0·8, the number of participants required to detect a difference in the mean response of matched pairs of 15 ml, with a crossover design was 10, based on a two-sided *α* of 0·05.

SPSS software version 27 (IBM SPSS statistics) and GraphPad Prism 9.0.0. (GraphPad Software Inc.) were used for statistical analysis and drawing graphs, respectively.

All results presented are mean and standard deviation unless otherwise stated. Normality of data was tested using Shapiro–Wilks test. Appropriate parametric tests were carried out on normally distributed data and equivalent non-parametric test when data were not normally distributed (i.e. upper gastrointestinal symptom scores).

For pH of gastric aspirate on insertion of the nasogastric tube, SBWC, GCV, SMA blood flux, blood glucose, serum insulin and gut hormones, baseline values were compared using the *t* test, to ensure that there was no carry over or sequence effect. Then change from baseline was calculated for each data point. These data were then graphically presented and used in subsequent analysis. The nature of the response, considering both magnitude and shape of each of the treatment curves, was compared using a two-way repeated-measures ANOVA (having ensured the conditions required for an ANOVA had not been violated), where factor 1 = feed type (F + ALG and F–ALG) and factor 2 = time point at which measurements were made. When an interaction was identified, simple effects of feed were explored by comparing the feeds at each time point applying a Bonferroni correction for multiple testing. When no statistically significant interaction was identified, the main effects of feed and time (applying Bonferroni corrections) were considered for statistical significance. Missing MRI values were derived by using the values either side of the missing value to calculate the ratio between the participant and the mean of the others and then applying this ratio to the mean value for the others at the missing timepoint'.

For subjective appetite scores, and the upper gastrointestinal symptom scores, the net incremental AUC (nAUC) was calculated by applying the trapezoid rule to data from which the baseline had been subtracted. The positive or negative sign of the number was preserved; hence, the AUC was considered to be net.

Energy consumed in the pasta meal, nAUC for subjective appetite scores, nAUC for the upper gastrointestinal symptom scores and end of the day upper gastrointestinal symptom scores were compared using *t* tests.

Differences were considered significant at *P* < 0·05 for all statistical tests.

## Results

### Baseline characteristics of participants

**Fig. 2.**
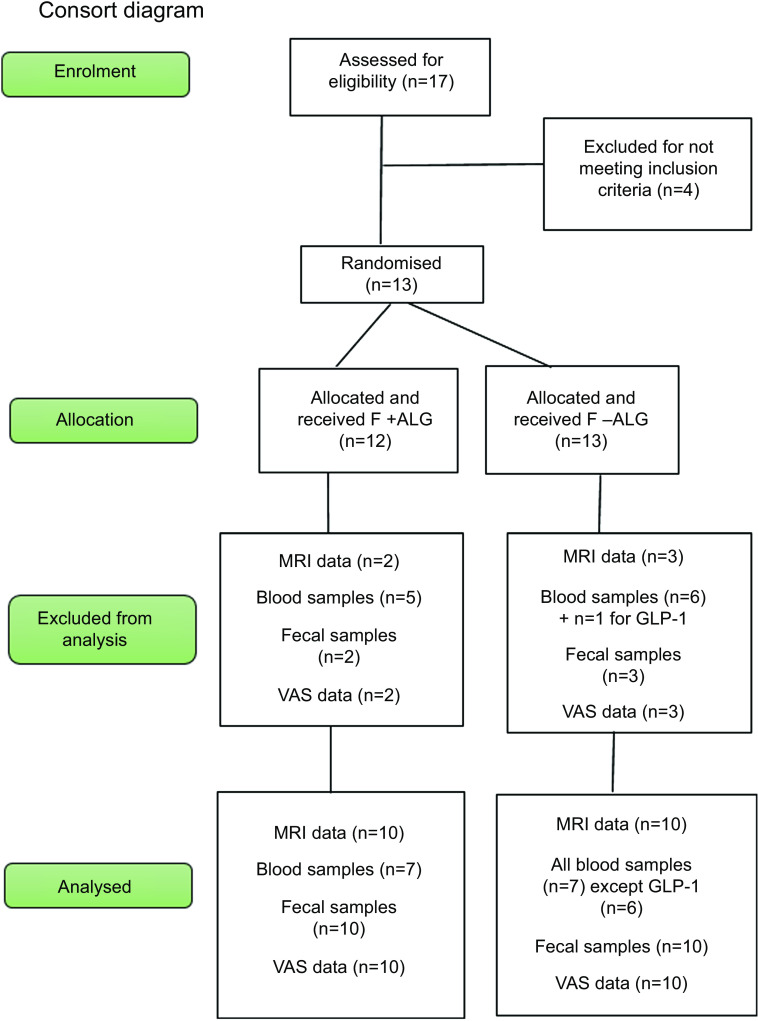
T2-weighted (TE = 300) axial images of the stomach showing characteristics of intragastric contents for the two feeds (F + ALG and F-ALG) 90 min after the commencement of feeding in one of the participants. The green arrows indicate the gastric contents. The freely mobile water appears as a bright white halo around a darker intragastric feed region which has a gel-like appearance, with F + ALG (left-hand image). The greyer appearance of the homogenous feed can be seen with F-ALG with no freely mobile water visualised as a bright white signal (right-hand image). F + ALG, alginate-containing feed; F–ALG, alginate-free feed; TE, echo time.

Thirteen participants meeting the inclusion criteria were randomised. One participant was withdrawn after 60 min, during the first arm of the study, due to vomiting. Of the remaining twelve participants, two were excluded subsequently from the analysis due to non-compliance with study procedure (*n* 1) or an abnormally high baseline GCV (*n* 1) (Fig. 2). The baseline characteristics of the remaining ten participants are presented in Table 2. In four participants, not all time points were available for blood samples. One participant voluntarily undertook a repeat trial, with blood collection, outside the MRI scanner. Blood sample analysis was thus undertaken on seven participants, except for GLP-1 as one participant was excluded due to an abnormally low, undetectable level. Results for six participants for GLP-1 were thus presented.

**Table 2.** Baseline characteristics of participants whose data were analysed (*n* 10)

Characteristic	Mean value	sd
Age (years)	28·6	6·6
Height (m)	1·8	0·08
Weight (kg)	78·51	8·6
BMI (kg/m^2^)	24·9	1·9
Waist circumference (cm)	87·8	2·9
BDI score	0·7	1·3
EAT score	4·4	3·3
Fasting blood glucose (mmol/l)	4·4	0·5
Number of bowel openings per d	2·0	0·7

BDI, Beck’s Depression Inventory; EAT, Eating Attitudes Test.

Gastric pH measured on insertion of the nasogastric tube was similar for the participants (*n* 10) before F + ALG and F-ALG (respectively, 3·5 ± 1·0 *v*. 3·4 ± 1·3, *P* = 0·83).

### Gastric contents characteristics

The T2-weighted images taken after the commencement of feeding revealed two distinct features in the stomach from 90 min until 120 min, one darker and non-homogenous, consistent with gel formation surrounded by a bright-white ring consistent with freely mobile water (F + ALG) and the other greyer and homogenously bright (F-ALG) consistent with a liquid state. Figure 1 shows an example of the MRI images seen.

### Small bowel water content volumes

**Fig. 3.**
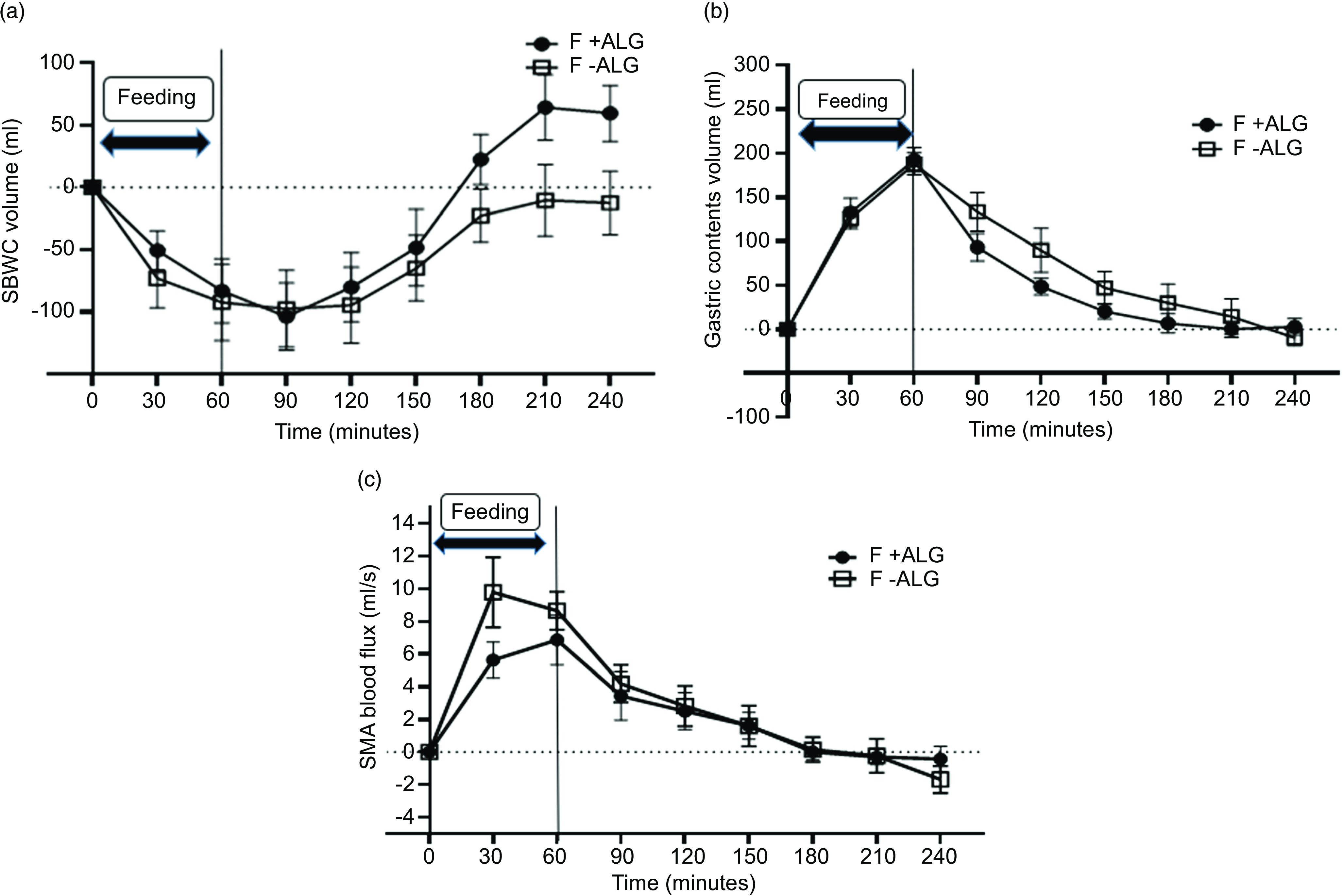
Change from baseline for small bowel water contents volume (SBWC) (a), gastric contents volume (GCV) (b) and superior mesenteric artery (SMA) blood flux (c) through 1 h of feeding and 3-h post-feeding (mean ± sem) in F + ALG and F-ALG (*n* 10). (a): For SBWC volume, no significant feed-by-time interaction was seen (*P* = 0·120). There was a statistically significant main effect for feed (*P* = 0·03) with the SBWC volume being greater for F + ALG compared with F-ALG and a significant main effect for time (*P* < 0·001). (b) For GCV, there was a statistically significant feed-by-time interaction (*P* = 0·04). Pairwise comparison between feeds revealed no significant difference in GCV for each of the time points (*P* > 0·05). (c): For SMA blood flux, there was no significant feed-by-time interaction (*P* = 0·22) or main effect of feed (*P* = 0·34), although there was a main effect of time (*P* < 0·001). F + ALG, alginate-containing feed; F–ALG, alginate-free feed.

Changes from baseline in SBWC are shown in Fig. 3(a). The values at baseline were similar (F + ALG = 119 ± 2 ml and F-ALG = 123 ± 3 ml; *P* = 0·65) and began to decrease on the commencement of feeding, reaching the lowest levels at 90 min F + ALG (–104 ± 86 ml) and F-ALG (–97 ± 98 ml), representing a decrease of 68 ± 28 % and 68 ± 30 % relative to baseline, respectively. The level then rose in both feeds, although the mean for the group for SBWC in the F-ALG remained below the baseline level at the end of the 3-h post-feeding period with the group mean for F + ALG above baseline values (Fig. 3(a)). There was, however, no significant feed-by-time interaction for the SBWC measurements (*P* = 0·12), although there were significant main effects of feed (*P* = 0·03) and time (*P* < 0·001) (data adjusted for baseline).

**Fig. 4.**
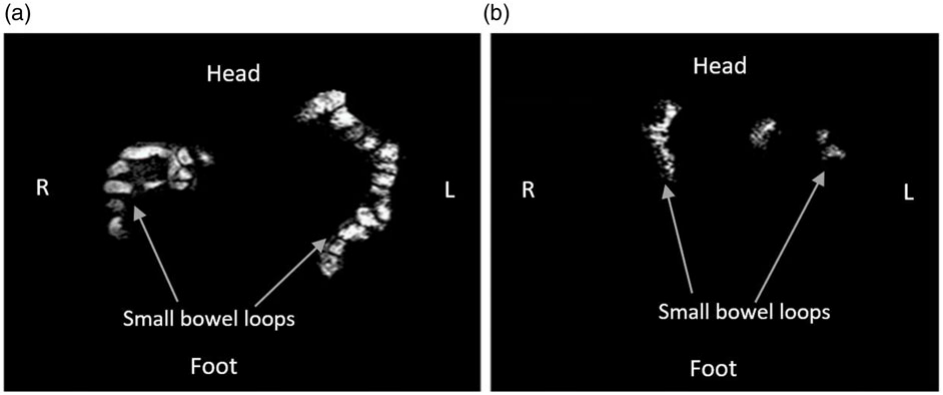
Coronal images of the abdomen in a single representative participant with the freely mobile water in the small bowel showing as bright white areas for F + ALG (a) and F-ALG (b), taken at 210 min after the commencement of feeding. More freely mobile water is evident with more bright white areas in A with F + ALG. (F + ALG, alginate-containing feed; F–ALG, alginate-free feed).

A representative image of the SBWC measurements in a single participant taken at 210-min post-feeding period demonstrates a larger irregular bright region in both flanks (Fig. 4(a)) for F + ALG which is consistent with higher SBWC volume and a smaller irregular bright area in both flanks (Fig. 4(b)) for F-ALG consistent with a smaller SBWC volume.

### Gastric contents volumes

Changes from baseline for GCV are shown in Fig. 3(b). The values at baseline were similar (F + ALG = 55 ± 7 ml and F-ALG = 68 ± 7 ml; *P* = 0·24) and then increased after feeding commenced, reaching absolute values of 248 ± 14 ml for F + ALG and 256 ± 9 ml for F-ALG at 60 min. Numerically, GCV then reduced more rapidly with the alginate-containing feed at each time point. Values had returned to baseline 3 h after feeding ceased in both groups. Less variation was seen in GCV profile between participants for F + ALG compared with F-ALG as indicated by the difference in magnitude of the error bars.

A significant feed-by-time interaction was seen (*P* = 0·04). Pairwise comparison, between feeds, at each time point identified no significant simple effects of feed.

### Superior mesenteric artery blood flux

Changes from baseline for SMA blood flux are shown in Fig. 3(c). The values at baseline were similar (F + ALG = 7·6 ± 0·6 ml/s and F-ALG = 8·5 ± 0·7 ml/s) (*P* = 0·24) and then increased once feeding commenced with a numerically earlier and higher peak with F-ALG (at 30 min, absolute values: 18·3 ± 6·8 ml/s) compared with F + ALG (at 60 min; absolute values: 14·4 ± 4·9 ml/s). Values had then returned to baseline by 180 min approximately.

No significant feed-by-time interaction (*P* = 0·22) or main effect of feed (*P* = 0·11) was seen, although there was a main effect of time (*P* < 0·001).

### Blood glucose

Blood glucose values at baseline, after an overnight fasting, were similar (F + ALG = 4·3 ± 0·3 mmol/l and F-ALG = 4·2 ± 0·3 mmol/l; *P* = 0·23). Numerically, after an initial drop from baseline at 10 min, an increase was seen in both feeds during the feeding period, resulting in absolute peaks at 60 min (F + ALG = 6·1 ± 0·5 mmol/l and F-ALG = 5·9 ± 0·4 mmol/l) followed by a gradual decline to baseline after 3 h (online Supplementary Item 3).

No significant feed-by-time interaction (*P* = 0·64) or main effect of feed (*P* = 0·97) was seen, whilst there was a significant main effect of time (*P* < 0·001).

### Serum insulin

**Fig. 5.**
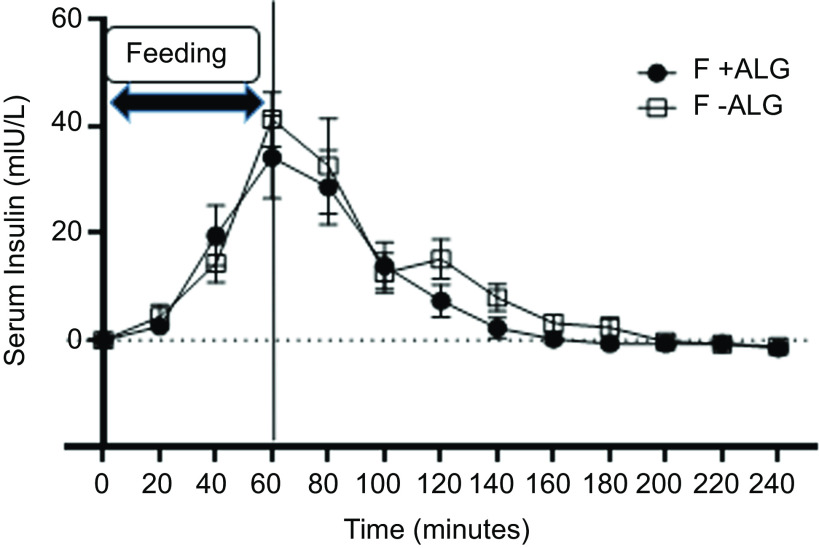
Change from baseline for serum insulin through 1 h of feeding and 3-h post-feeding (mean ± sem) in F + ALG and F-ALG feeds (*n* 7). No significant feed-by-time interaction (*P* = 0·69) was seen, but there was a significant main effect of feed (*P* = 0·01) and time (*P* < 0·001). (F + ALG, alginate-containing feed; F–ALG, alginate-free feed).

Changes from baseline are shown for serum insulin in Fig. 5. Serum insulin levels at baseline were similar (F + ALG = 7·5 ± 1·2 mIU/l and F-ALG = 6·5 ± 0·7 mIU/l; *P* = 0·31). Serum insulin levels then rose after feeding commenced, reaching peak levels at 60 min in F + ALG (41·8 ± 22·7 mIU/l) and F-ALG (47·9 ± 14·1 mIU/l), and then numerically, returning to baseline by 160 min in F + ALG and 200 min in F-ALG feeds.

No significant feed-by-time interaction was seen for serum insulin levels (*P* = 0·68), although significant main effects of feed (*P* = 0·01) and time (*P* < 0·001) were observed.

### Serum total glucose-dependent insulinotropic polypeptide

**Fig. 6.**
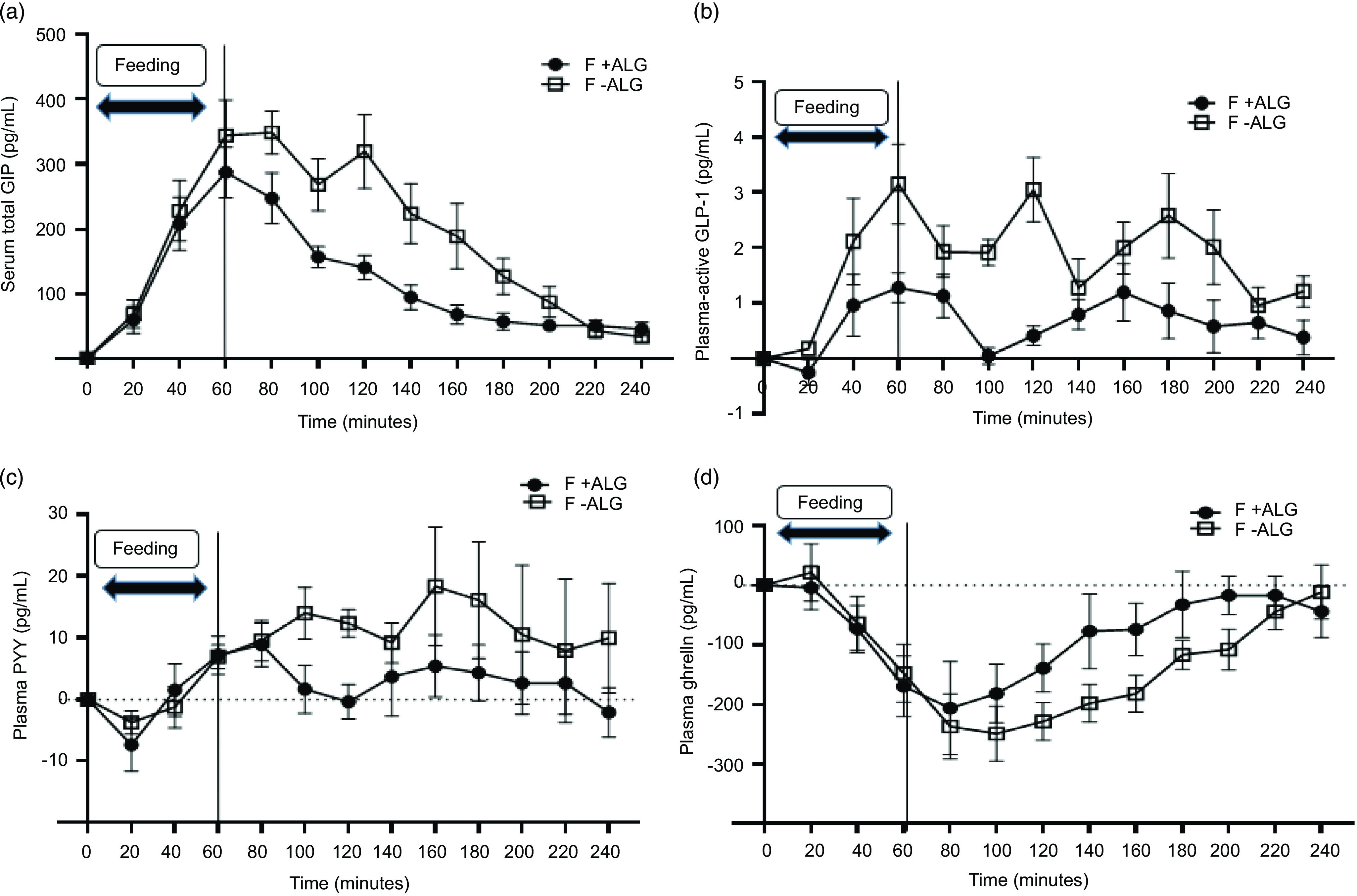
Change from baseline for (a) serum total GIP (*n* 7), (b) plasma-active GLP-1 (*n* 6), (c) plasma PYY (*n* 7) and (d) plasma ghrelin (*n* 7), through 1 h of feeding and 3-h post-feeding (mean ± sem) for F + ALG and F-ALG. (a) (serum total GIP): There was a significant feed-by-time interaction (*P* = 0·002). Pairwise comparison showed significantly lower levels with F + ALG than with F-ALG at 100 min (*P* = 0·04), 120 min (*P* = 0·01), 140 min (*P* = 0·03), 160 min (*P* = 0·03) and 180 min (*P* = 0·03). (b) (plasma-active GLP-1): There was a significant feed-by-time interaction (*P* = 0·02). Pairwise comparison showed significantly lower levels with F + ALG than with F-ALG at 60 min (*P* = 0·03), 100 min (*P* = 0·001), 120 min (*P* = 0·002) and 180 min (*P* = 0·003). (c) (plasma PYY): No feed-by-time interaction (*P* = 0·44) was seen, but a main effect of feed (*P* = 0·004) was observed with no significant main effect of time (*P* = 0·10). (d) (plasma ghrelin): There was a tendency for a feed-by-time interaction observed (*P* = 0·07), no significant effect of feed (*P* = 0·30), but a significant main effect of time was observed (*P* < 0·001). (F + ALG, alginate-containing feed; F–ALG, alginate-free feed). GIP, glucose-dependent insulinotropic polypeptide; GLP-1, glucagon-like-peptide 1; PYY, peptide YY.

Changes from baseline for serum total GIP are shown in Fig. 6(a). The values at baseline were similar (F + ALG = 35·9 ± 20·2 pg/ml and F-ALG = 37·2 ± 19·3 pg/ml; *P* = 0·914) and then increased once feeding commenced, reaching absolute peak values at 1 h (F + ALG = 323·0 ± 91·6 pg/ml and F-ALG = 381·6 ± 150·0 pg/ml). Numerically, F + ALG then declined more rapidly, and with both feeds, levels approached the baseline by 240 min.

A significant feed-by-time interaction was seen (*P* = 0·002). On exploring simple effects, significantly lower levels were seen with F + ALG than with F-ALG at 100 min (*P* = 0·04), 120 min (*P* = 0·01), 140 min (*P* = 0·03), 160 min (*P* = 0·03) and 180 min (*P* = 0·03).

### Plasma-active glucagon-like-peptide 1

Changes from baseline for plasma GLP-1 level are shown in Fig. 6(b). The values at baseline were similar (F + ALG = 3·4 ± 3·8 pg/ml and F-ALG = 3·8 ± 6·1 pg/ml) (*P* > 0·05)) and then increased once feeding commenced. Numerically, F + ALG showed a more blunted response appearing to return to baseline by 100 min, increase again and then return almost to baseline 3-h post-feeding. In contrast, numerically F-ALG showed a greater response and then oscillated at a higher level.

A significant feed-by-time interaction was seen (*P* = 0·02). On exploring simple effects, significantly lower levels were seen with F + ALG than with F-ALG at 60 min (*P* = 0·03), at 100 min (P = 0·001), at 120 min (*P* = 0·002) and at 180 min (*P* = 0·003).

### Plasma peptide YY

Changes from baseline for plasma PYY levels are shown in Fig. 6(c). The values at baseline were similar (F + ALG = 74·4 ± 11·7 ml and F-ALG = 82·3 ± 7·7 ml; *P* = 0·52) briefly decreased once feeding commenced and then began to rise above baseline by 60 min. Numerically, F + ALG then returned to baseline by 120 min before demonstrating a blunted response compared with F-ALG which remained above baseline at 240 min.

No significant feed-by-time interaction was seen (*P* = 0·44); there was a significant main effect of feed (*P* = 0·004) with F-ALG being higher, and no main effect of time (*P* = 0·10).

### Plasma ghrelin

Changes from baseline for plasma levels of ghrelin are shown in Fig. 6(d). The values at baseline were similar (F + ALG = 992·6 ± 354·0 pg/ml and F-ALG = 985·0 ± 222·8 pg/ml; *P* = 0·90). Once feeding commenced, numerically both levels decreased until 80 min with F + ALG and 100 min with F-ALG; with both the level returning to baseline by 240 min in both feeds.

There was a tendency for a significant feed-by-time interaction (*P* = 0·07), no significant main effect of feed (*P* = 0·3); there was a significant main effect of time (*P* < 0·001).

### Appetitive VAS and pasta meal consumption

The time courses for appetitive VAS across the hour of feeding and 3-h postprandial are available in Supplementary Item 4. The difference in nAUC between F + ALG and F–ALG for ratings of hunger, satisfaction, fullness, desire to eat and prospective food consumption across the study were not significant (online Supplementary Item 5). Mean energy intake from the pasta meal, 3 h after 300 ml of enteral feed had been given was 7018·5 kJ ± 479·1 (F + ALG) and 8228·8 kJ ± 519·8 (F-ALG). The difference showed a tendency for a lower energy intake after F + ALG compared with F-ALG (*P* = 0·06).

### Upper gastrointestinal symptom scores

The time courses for upper gastrointestinal symptom scores across the hour of feeding and 3-h postprandial are available in Supplementary Item 6. The differences in nAUC between F + ALG and F-ALG in reported upper gastrointestinal symptom scores from baseline to the end of the 3-h post-feeding period were not statistically significant (online Supplementary Item 7). Similarly, no statistically significant difference was seen between the two feeds when these symptoms were assessed on the day of intervention at bedtime (online Supplementary Item 8).

## Discussion

The purpose of this study was to investigate in healthy participants whether acute exposure to an NGTF that forms an intragastric gel results in different gastrointestinal responses compared with a non-gelling feed. Using MRI, we demonstrated with the alginate-containing feed an intragastric appearance consistent with gelling, which previously has been shown only in animal studies^(32)^ or in human studies following oral consumption^(53–55)^. Whilst necessarily cautious in our interpretation of the visual appearance of the MRI images of the stomach contents as ‘consistent’ with gelling (given that the stomach contents were not observed directly) previous *in vitro* work (Supplementary Item 9) in which we simulated the acidic gastric environment, supports our conclusions. We noted differences in GCV and SBWC response between the two feeds.

GCV reduced more rapidly after F + ALG and individual GCV responses appeared less varied. The gut peptides GLP-1, GIP and PYY, which slow gastric emptying^(56)^, increased as expected on initiation of feeding. Peripherally, GLP-1 is secreted from the intestinal L-cells following sensing of glucose and fat in the small bowel, although gastric distension also activates GLP-1-containing neurones in the nucleus of the solitary tract^(56,57)^. Initial gastric emptying occurs more rapidly (a shorter lag time) with liquids, compared with solids, of equivalent energy and macronutrient content^(14,53)^ and is slowed marginally by greater viscosity^(53,54)^. A nutrient poor liquid with low viscosity would be expected to leave the stomach more rapidly than the more viscous and nutrient-dense comparator feed, or a more viscous and nutrient-dense gel component. The halo of intragastric freely mobile liquid around the gel may have contributed to the more rapid reduction in GCV if of low viscosity and nutrient-poor, potentially due to selective nutrient retention by the gel, or if the gel resisted mixing with the less nutritive gastric secretions. The blunted gut peptide response would be consistent with a lower nutritive value liquid and would further perpetuate the more rapid reduction in GCV. Others have shown ‘sieving’ with a mixed consistency meal, resulting in rapid gastric emptying of a nutrient-poor liquid, compared with a soup with the solid and liquid components combined^(13)^. The contrast in texture, due to our study design, was less marked than previous studies, but some sieving may have occurred between the gel and liquid. The more rapid reduction in GCV, hence faster reduction in gastric distension, may contribute to a reduced risk of oesophageal reflux^(24)^.

The similar levels of circulating glucose and serum insulin with the two feeds, in contrast to the blunted incretins GIP and GLP-1 with F + ALG, would refute a marked difference in the rate of delivery of at least glucose, to the small intestine. Similarly, no difference was seen in blood glucose profile when an alginate-containing drink was consumed orally^(55)^. Furthermore, in our study, a similar SMA blood flux was seen with the two feeds, suggesting comparable stimulation with respect to glucose, at least^(42)^.

Alternatively, alginate reaching the small intestine may mask nutrient sensing if the gelling fibres interact with the intestinal mucus layer^(58)^ but appear not to mask the nutrients (e.g. glucose), with respect to stimulating SMA blood flux^(42)^ or prevent absorption. An *in vitro* ‘model gut’ might be used to establish whether there is selective retention of nutrients within the alginate gel^(59)^.

We believe this study is the first to identify a difference in SBWC with an alginate-containing NGTF compared with a non-gelling feed. The initial reduction, below baseline with both feeds, in SBWC was comparable to other studies, including a comparison of bolus and continuous tube feeding^(49,60)^ and may reflect an anticipatory increased absorption (despite bypassing the cephalic phase)^(61)^; early nutrient delivery^(49)^ which would be consistent with the numerical increase in SMA blood flux^(42)^; and early gastroileal reflex activation promoting distal ileal emptying into the ascending colon^(49)^.

The subsequent progressive increase in SBWC, with both feeds, has been attributed to pancreato-biliary and enterocyte secretions in the ‘intestinal phase’ combined with greater movement of mobile water from the stomach^(49)^. The numerically greater rebound, above baseline, after 180 min, with F + ALG is exemplified by more ‘bright-white’ freely mobile water in the MRI images at 210 min. Gel dissolving in the higher pH of the small bowel may have released bound water, or the blunting of the postprandial secretion of PYY, following distal small bowel nutrient sensing^(56)^, may have contributed to the difference with the alginate feed. PYY is a major pro-absorptive hormone^(56,62,63)^ and influences SBWC by strongly inhibiting gastrointestinal motility, and pancreatic, gastric, and small bowel secretions^(56)^. PYY also triggers the ‘ileal brake’; hence, it slows intestinal transit, increasing half-time available for water absorption within the small intestine^(56,64)^. The clinical impact of the small difference in SBWC between the two feeds (maximum difference approximately 60 ml at 210 min and 240 min) seen in our study might be explored by measuring colonic water using a validated MRI technique^(65)^ whilst concurrently quantifying stool consistency in healthy and patient groups who were chronically administered a NGTF.

The relatively small volume of feed given may have reduced the effect on appetite in contrast to other studies^(54)^ where addition of alginate increased satiation, although the oral route had been used^(54)^. Absence of a marked difference in energy intake from the pasta meal was not unexpected, given that ghrelin levels had returned to baseline with both feeds prior to consumption of the pasta.

Compliance with the upper gastrointestinal symptom scores recording and completion of the fecal charts, and fecal sample collection was high and gave no evidence of changed or abnormal bowel habits or symptoms with either feed. This may reflect the young, healthy nature of the participants and the small amount of feed provided.

The feeds had comparable energy, macronutrients, osmolarity and fibre content. The different fibre sources, however, may have confounded our interpretation of the results. The Scientific Advisory Committee on Nutrition (SACN, 2015) defines dietary fibre as including all carbohydrates that are neither digested nor absorbed in the small intestine and which have a degree of polymerisation of three or more monomeric units, plus lignin^(66)^. Different dietary fibres, however, exhibit diverse chemical and physical properties^(66,67)^, including the production of SCFA on fermentation in the colon, and may impact gastric emptying, SBWC, glycaemic and appetitive responses^(33,68–71)^. The acute nature of our study (over 4 h) and the single exposure to each feed (hence precluding microbial changes seen with prebiotics which stabilise over 6 weeks^(67)^) were intended to limit the impact of any differences in fermentation and SCFA production, particularly in the earlier stages of the study, between the two feeds. Moreover, in our study, the SBWC profile for the inulin containing comparator feed was similar to that seen in the fibre-free arm of a previous study^(60)^, although the return to baseline 240 min was less marked. A sufficiently stable feed that was identical, apart from the absence of alginate, was not available to us, due to production challenges but would benefit future studies.

We acknowledge that GCV is a proxy for gastric emptying, as it includes gastric secretions and rate of delivery of feed into the stomach (the latter identical in the two feeds in this study by design) as well as passage of gastric contents out of the stomach. Differences in gastric secretions are thought to be unlikely between the two feeds as the energy and macronutrient content were the same and delivery volume is considered to be more important than content with respect to gastric responses^(57)^. Calculation of gastric emptying rate and time to 50 % of emptying were not considered valid, because the feed was delivered over a 60-min period rather than as a discrete bolus.

Difficulties with cannulation resulted in a smaller sample than intended for the blood-based measurements. This may have resulted in an increased risk of type 2 errors. In future studies, more participants should be recruited in anticipation of this issue.

### Conclusions

In this study using MRI, we have demonstrated that a NGTF containing alginate results in an intragastric appearance consistent with the development of a gel surrounded by a halo of freely mobile water. Differences in gastrointestinal response, including SBWC and GCV, were demonstrated between this feed and a non-gelling feed. The stability, however, of these differences over a longer feeding period and their clinical relevance are not fully understood. Future studies are required over a longer time, and in clinical populations using a gelling, alginate-containing feed and a matched alginate-free feed.

## Supplementary material

For supplementary material accompanying this paper visit https://doi.org/10.1017/S0007114523000302. click here to view supplementary material
