# Supplementary material for: A comparative, randomised MRI study of the physiological and appetitive responses to gelling (alginate) and non-gelling nasogastric tube feeds in healthy men
Source: Br J Nutr. 2023 Feb 7;130(8):1316–28. doi: 10.1017/S0007114523000302 (PMC10511685; doi:10.1017/S0007114523000302)
Supplement: Supplementary file 1 [file S0007114523000302sup001.docx]

**Supplementary material**

**Supplementary Item 1: Feed composition**

**
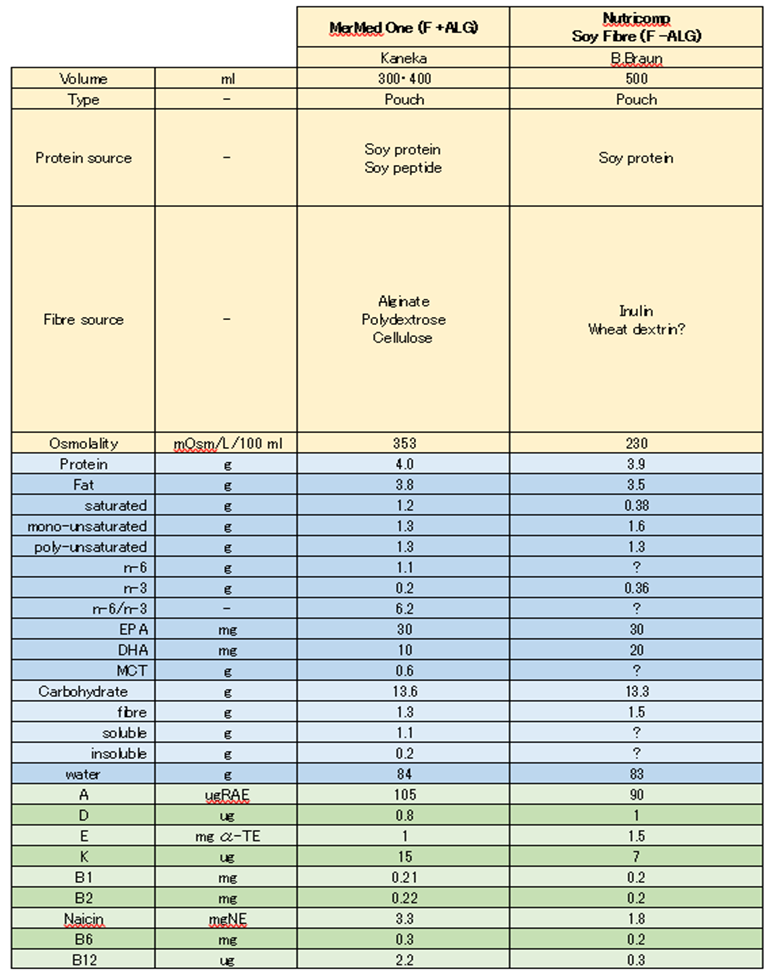
**

**Supplementary Item 2: Protocol for preparation of the pasta meal**

The pasta meal was prepared in individual portions the night before it was required for a study. For every 125 g of dry pasta, 800ml of boiling water was added and then microwaved on full power for 7 min, mixed and then microwaved for a further 6 min. The cooked pasta was then drained in a colander and rinsed with cold water. Three portions of the pasta were then transferred into a deep glass bowl when cold and stored in a fridge at 4^o^C overnight. In the morning, 510 g of pasta sauce (tomato and Basil pasta sauce, Napolina), 120 g (40 g x 3) of pre-grated mature British Cheddar cheese (Sainsbury’s), and 60 mL (20 mL x3) of olive oil composed of refined olive oils and virgin olive oils (Sainsbury’s) were added and mixed well in the glass bowl. Immediately prior to serving the pasta and sauce was divided into three bowls equally, and a portion was microwaved for 4 min, and then heated for further 30 second periods as required, until heated throughout to a temperature of ≥82°C (Food Standards Regulation 2007). The pasta meal was then allowed to cool down to 50°C before being served to the participant. The other two portions were similarly heated, as required.

**Supplementary Item 3: Time course relative to baseline for blood glucose**

**
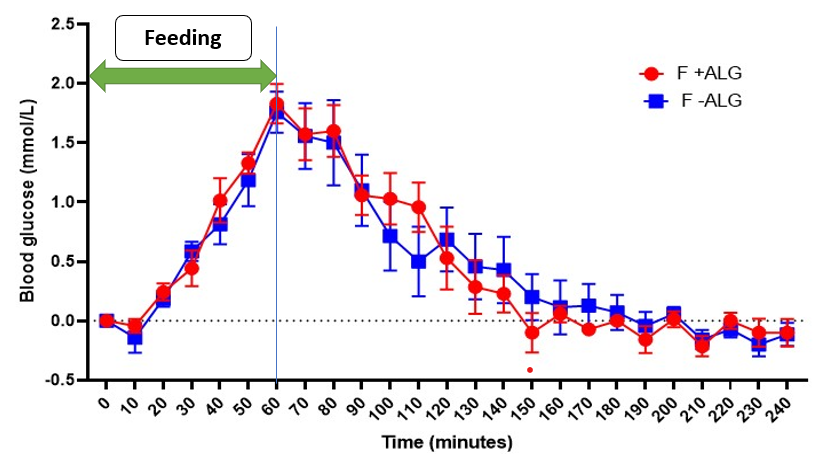
**

Change from baseline for blood glucose through one hour of feeding and three hours post feeding (mean ±SEM) in F+ALG and F-ALG (n=7). No significant feed by time interaction (P= 0.64) or main effect of feed (P=0.97) was seen, although there was a significant main effect of time (P<0.001).

**Supplementary Item 4: Time courses for appetitive visual analogue scores**

Appetitive scores from baseline to 233 min in F+ALG and F-ALG feeds (n=10). Values represent mean and SEM for hunger, satisfaction, fullness, desire to eat and prospective food consumption.


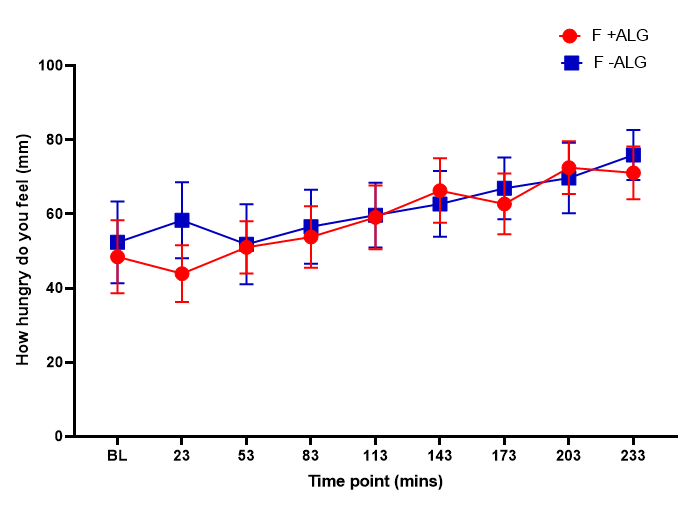


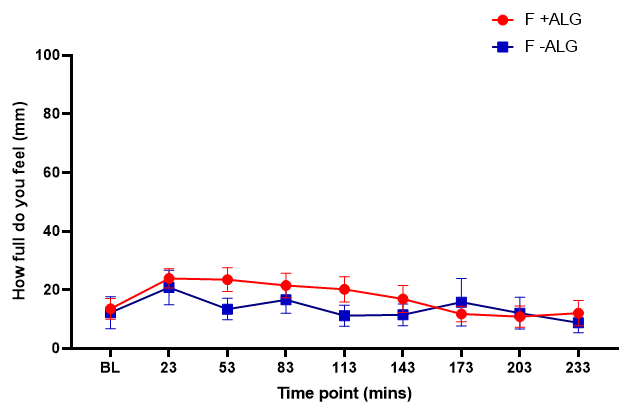


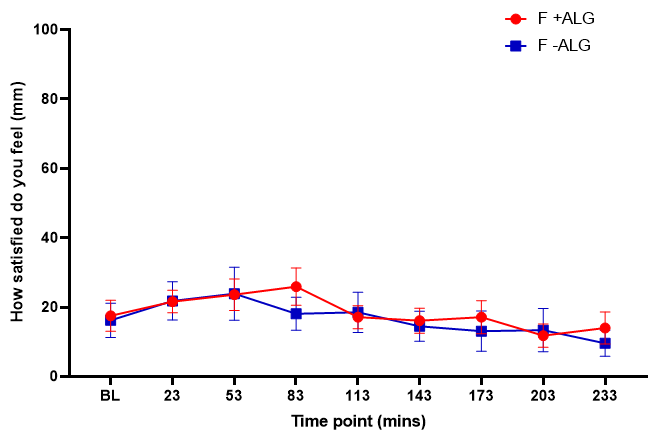


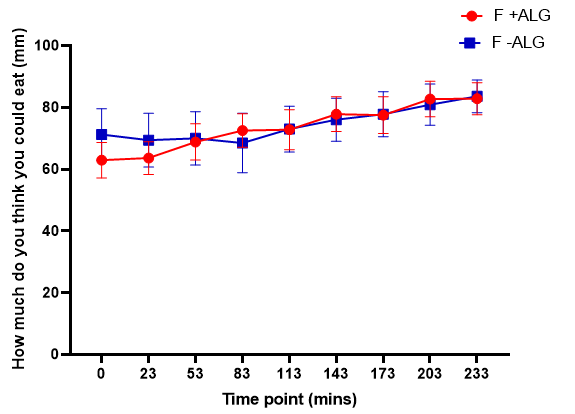


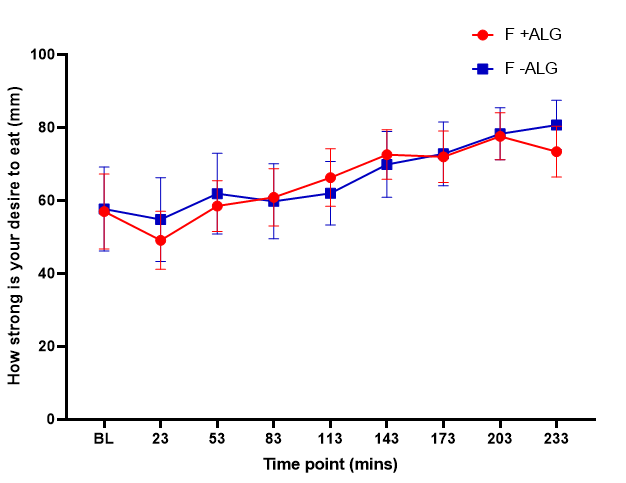


**Supplementary Item 5:**

| **Item 5: Net area under the curve (nAUC) from baseline to 233 min for the appetite ratings. (n=10).** | | | | | |
| --- | --- | --- | --- | --- | --- |
| **Appetite scores** | **F +ALG** | | **F -ALG** | | **P-value^*^** |
|  | **Mean** | **SEM** | **Mean** | **SEM** |  |
| **Hunger (mm.min over 4 hours)** | 2746 | 1212 | 2515 | 1033 | P=0.98 |
| **Satisfaction (mm.min over 4 hours)** | 539 | 881 | 1406 | 1243 | P=0.91 |
| **Fullness (mm.min over 4 hours)** | 1227 | 729 | 1210 | 922 | P=0.48 |
| **Desire (mm.min over 4 hours)** | 2164 | 920 | 1001 | 1270 | P=0.81 |
| **Prospective food consumption (mm.min over 4 hours)** | 2528 | 932 | -317 | 1192 | P=0.20 |

SEM, standard error of the mean

*- P values for paired T- test comparing F +ALG with F –ALG.

**Supplementary Item 6: Time courses for Upper Gastrointestinal symptom scores**

Upper gastrointestinal symptom scores from baseline to 233 min in F+ALG and F-ALG feeds (n=9).


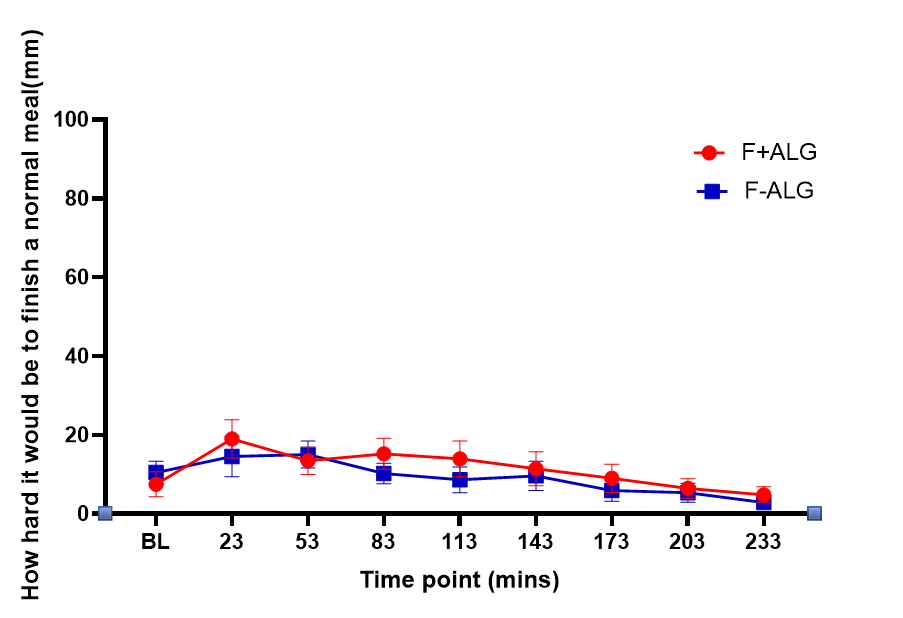


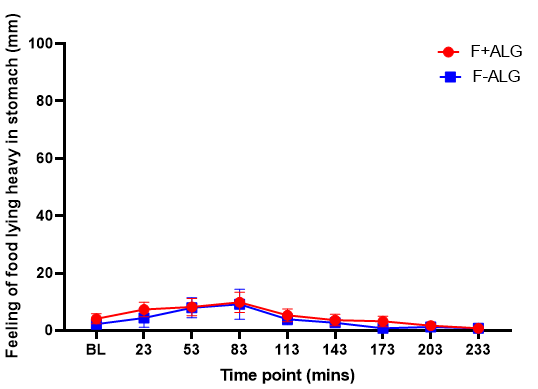


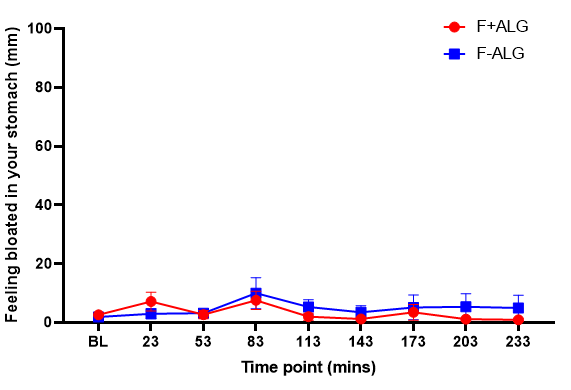


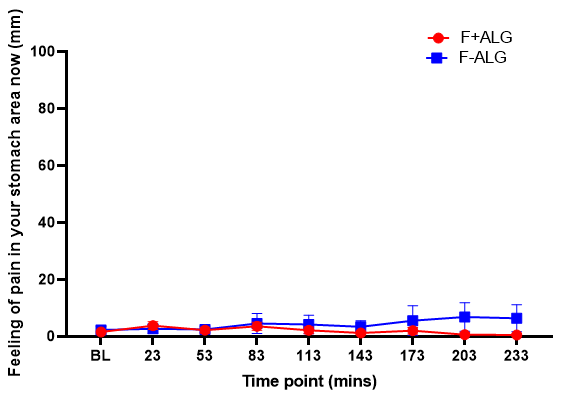


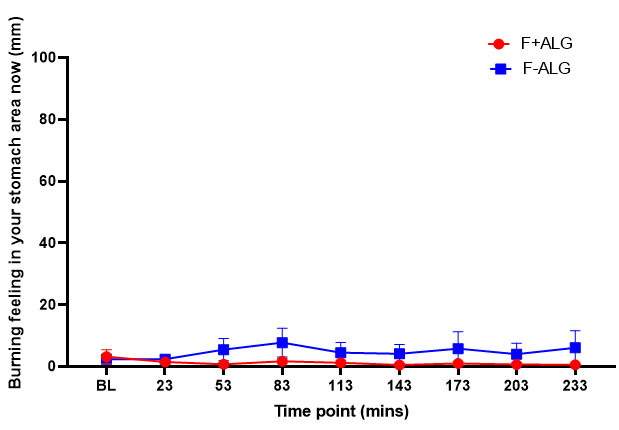


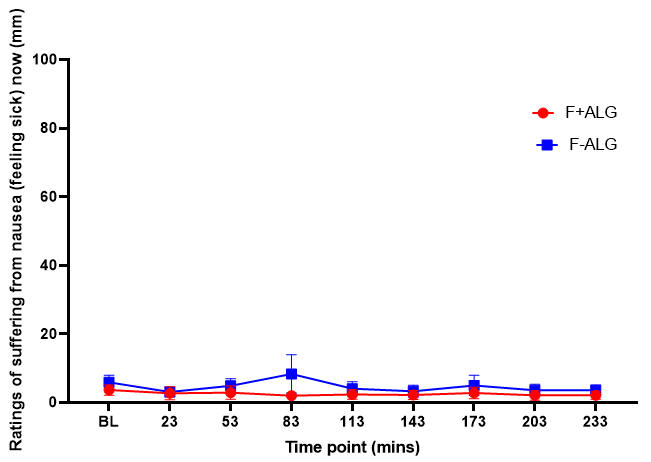


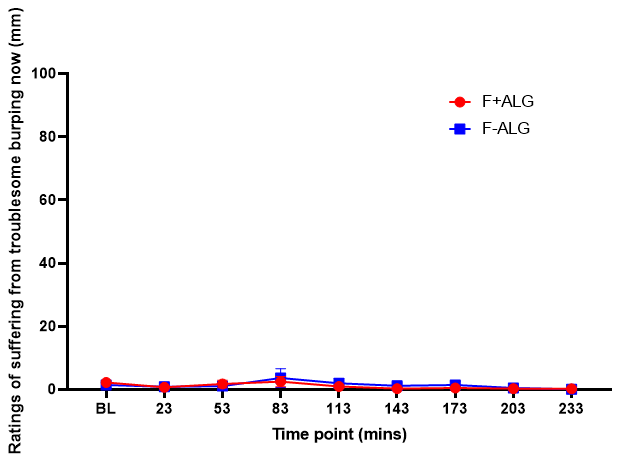


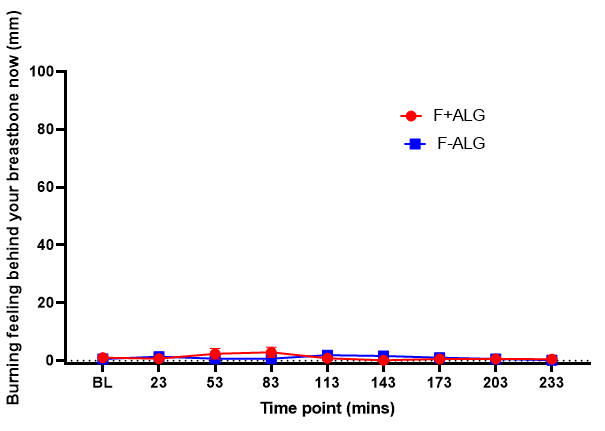


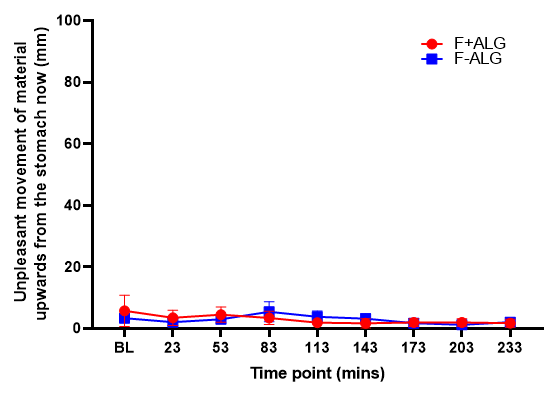


F+ALG, alginate containing feed

F-ALG, alginate free feed

**Supplementary Item 7**

| **Item 7: Net areas under the curve (nAUC) for all the upper gastrointestinal symptoms from baseline to 233min (n=9)** | | | | | |
| --- | --- | --- | --- | --- | --- |
| **Upper GI symptoms** | **F +ALG (mm.min over 4 hours)** | | **F -ALG (mm.min over 4 hours)** | |  |
|  | **Mean** | **Interquartile range** | **Mean** | **Interquartile range** | **P value** |
| **1. How hard it would be to finish a normal meal?** | 1116 | 2977 | -273 | 2808 | P=0.18^a^ |
| **2. How bad was feeling of food lying heavy in stomach?** | 442 | 839 | 366 | 581 | P=0.14^b^ |
| **3. How much are you suffering from feeling bloated in your stomach?** | 205 | 693 | 219 | 988 | P=0.28^a^ |
| **4. How much are you suffering from pain in your stomach area now?** | 137 | 597 | 76 | 321 | P=0.54^a^ |
| **5. How much are you suffering from a burning feeling in your stomach area now?** | -515 | 241 | -58 | 484 | P=0.59^b^ |
| **6. How much are you suffering from nausea (feeling sick) now?** | -10 | 339 | -217 | 1081 | P=0.49^a^ |
| **7. How much are you suffering from troublesome burping now?** | -268 | 321 | 5 | 223 | P=1.00^b^ |
| **8. How much are you suffering from a burning feeling behind your breastbone now?** | 25 | 348 | 103 | 34 | P=0.59^b^ |
| **9. How much do you taste acid in your mouth now?** | 519 | 363 | -163 | 76 | P=0.33^b^ |
| **10. How much are you feeling an unpleasant movement of material upwards from the stomach now?** | -737 | 203 | 21 | 974 | P=0.95^b^ |

SEM, standard error of the mean

^a^- P value for T- test

^b^- P value for Wilcoxon ranked test.

**Supplementary Item 8**

| **Item 8: Upper gastrointestinal symptom scores just before going to bed on the day participants received the NGTF feeds. (n=9).** | | | | | |
| --- | --- | --- | --- | --- | --- |
| **Upper GI symptoms** | **F +ALG (mm)** | | **F -ALG (mm)** | | **P value** |
|  | Mean | SEM | Mean | SEM |  |
| **How hard it would be to finish a normal meal** | 51 | 10 | 58 | 9 | P=0.19^a^ |
| **How bad was feeling of food lying heavy in stomach** | 33 | 9 | 40 | 10 | P=0.19^a^ |
| **Feeling of being bloated in stomach** | 8 | 4 | 15 | 7 | P=0.35^b^ |
| **Degree of pain in stomach** | 2 | 1 | 5 | 9 | P=0.25^b^ |
| **Burning feeling in the stomach** | 2 | 1 | 4 | 8 | P=0.20^b^ |
| **Feeling of nausea** | 7 | 5 | 4 | 9 | P=0.49^b^ |
| **Feeling of troublesome burping** | 5 | 2 | 2 | 3 | P=0.66^b^ |
| **Burning feeling behind the breastbone** | 5 | 3 | 2 | 5 | P=1.00^b^ |
| **Feeling of acid taste in mouth** | 3 | 2 | 2 | 5 | P=0.72^b^ |
| **Unpleasant movement of material upwards from stomach** | 5 | 3 | 3 | 6 | P=0.72^b^ |

SEM, standard error of the mean

^a^- P value for T- test

^b^- P value for Wilcoxon ranked test.

**Supplementary Item 9**

**Item 9A**


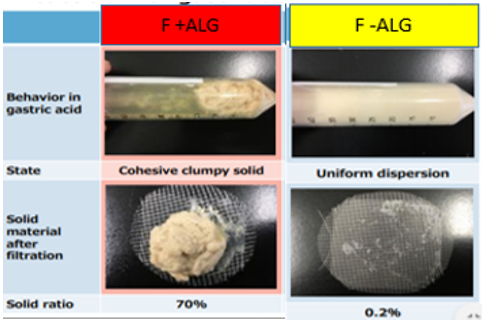


**Item 9A: In vitro characterization of the physical behaviour of the INTERVENTION feed (F+ALG) (left) and the COMPARATOR feed (F-ALG) (right) after addition of simulated gastric acid. The procedure was carried out by the Kaneka Corporation laboratory in Japan.**


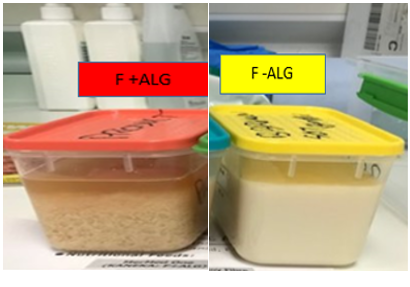


**Item 9B**

**Item 9B: In vitro characterization of the gelling behaviour of the INTERVENTION feed (F+ALG) (left) and the COMPARATOR feed (F-ALG) (right) after addition of simulated gastric acid prior to MRI scanning (Item 9c). The procedure was carried out at the Sir Peter Mansfield Imaging Centre, University of Nottingham.**

**
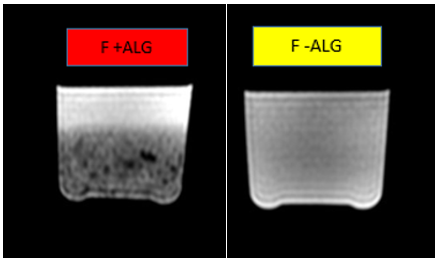
**

**Item 9C**

**Item 9C: In vitro MRI images of the INTERVENTION feed (F+ALG) (left) and the COMPARATOR feed (F-ALG) (right) (shown in Item 9b) after addition of simulated gastric acid. The procedure was conducted at the Sir Peter Mansfield Imaging centre.**

**In Items 9A and 9B consistently greater gelling can be seen in the INTERVENTION feed (F+ALG) (left) compared with the COMPARATOR feed (F-ALG) (right), on the addition of simulated gastric acid. Consequently, differences are seen between the two feeds in the appearance of the MRI images (Image 9C), created by MRI scanning the samples presented in Image 9B. (**F+ALG, alginate containing feed; F-ALG, alginate free feed)
